# Editorial: Biomarkers for progressive pulmonary fibrosis

**DOI:** 10.3389/fmed.2025.1595959

**Published:** 2025-04-08

**Authors:** Alexandre Todorovic Fabro, Luka Brcic, Rosane Duarte Achcar

**Affiliations:** ^1^Department of Pathology and Legal Medicine, Ribeirão Preto Medical School, University of São Paulo, Ribeirão Preto, Brazil; ^2^Hospital Graz II, Graz and Medical University of Vienna, Vienna, Austria; ^3^Department of Medicine, National Jewish Health, Pathology Division, Denver, CO, United States

**Keywords:** progressive pulmonary disease, interstitial lung diseases, biomarkers, lung diseases - pathology, molecular diagnosis

## Introduction

Progressive pulmonary fibrosis (PPF) presents a significant challenge in respiratory medicine, characterized by disease progression despite conventional treatment, extending beyond idiopathic pulmonary fibrosis and encompassing a broad spectrum of interstitial lung diseases (ILDs). Identifying reliable biomarkers to predict disease progression, monitor response to therapy, and guide clinical decision-making remains an urgent unmet need. In this Research Topic, “*Biomarkers for Progressive Pulmonary Fibrosis*,” we present a collection of seven articles exploring innovative biomarker discovery approaches, spanning molecular, metabolic, and imaging-based methodologies.

## Exploring the biomarker for PPF

A key contribution in this Research Topic is the study by Ge et al., which introduces the *blood urea nitrogen-to-albumin ratio (BAR)* as a prognostic indicator for 1-year mortality in idiopathic pulmonary fibrosis (IPF) patients. Their findings suggest that BAR, a simple and cost-effective biomarker, could serve as a useful clinical tool for early risk stratification. Similarly, Fan et al. provide a comprehensive analysis of risk factors for PPF in ILD, proposing a prognostic nomogram incorporating clinical and imaging data to predict disease progression with high accuracy.

At the molecular level, Lavis et al. highlight the role of fibroblast activation protein alpha (FAPα) as a promising biomarker for fibrotic progression. The study discusses how FAPα detection in human fluids and imaging modalities could enhance the early diagnosis and monitoring of PPF. Complementing this, Wang et al. explore the intricate crosstalk between *TGF-*β *signaling and microRNAs*, elucidating the regulatory mechanisms underlying fibrosis progression and identifying potential therapeutic targets.

In the domain of metabolic profiling, Zhang et al. use a metabolomics-based approach to investigate the metabolic alterations in a murine model of IPF. Their findings show that the dysregulation of lipid metabolism and oxidative pathways are key contributors to fibrogenesis, introducing the way for novel biomarker discovery in clinical settings. Expanding on therapeutic strategies, Lin et al. demonstrate that inhibition of *PCSK9*—a key regulator of cholesterol metabolism—mitigates pulmonary hypertension secondary to fibrosis by modulating epithelial-mesenchymal transition and Wnt/β-catenin signaling, opening new possibilities for targeted treatment.

Finally, Min et al. provide a bibliometric and visual analysis of macrophage-related research in pulmonary fibrosis. Their study not only maps the evolving research landscape but also identifies key molecular targets and signaling pathways involved in macrophage-driven fibrosis.

## Future directions and perspectives

Despite significant advances in biomarker discovery for PPF, several challenges remain in their clinical validation. Future research should focus on validating promising biomarkers, such as the blood urea nitrogen-to-albumin ratio (BAR), fibroblast activation protein alpha (FAPα), and metabolomic signatures, in large, multicenter cohorts. The integration of these biomarkers into routine clinical practice requires standardized protocols and clinical trials to ensure their reliability and reproducibility across diverse patient populations.

Additionally, the development of biomarker-based predictive models, such as prognostic nomograms incorporating molecular and imaging data, holds promise for personalized disease management. Combining multi-omics approaches—including genomics, transcriptomics, proteomics, and metabolomics—could enhance our understanding of fibrosis progression and identify novel therapeutic targets.

The targeted therapeutic interventions, such as PCSK9 inhibitors and modulators of TGF-β signaling, suggests that biomarker-driven treatment strategies may soon be feasible. The next steps should include clinical trials designed to evaluate biomarker-guided therapeutic decisions, optimizing treatment selection and improving patient outcomes.

Ultimately, the successful integration of these findings into clinical practice will require interdisciplinary collaboration among pulmonologists, pathologists, radiologists and scientists. Artificial intelligence and machine learning tools will likely play a crucial role in analyzing complex biomarker datasets and refining risk stratification models.

## Conclusions

Biomarker discovery for PPF has made significant new insights into disease pathogenesis and potential therapeutic targets. This Research Topic of studies highlights the importance of integrating molecular, metabolic, and imaging biomarkers to improve early diagnosis, risk assessment, and treatment strategies.

While promising biomarkers, such as BAR, FAPα, and metabolic profiles, have shown potential in identifying disease progression, their clinical implementation remains challenging. Standardized validation, multicenter studies, and interdisciplinary approaches will be essential to translating these findings into practical applications.

The future of PPF management lies in precision medicine, where biomarker-guided strategies enable personalized therapeutic interventions. By leveraging multi-omics data, predictive modeling, and emerging treatment options, clinicians may soon have more effective tools to combat disease progression. Continued collaboration and innovation in this field will be key to improving outcomes for patients with progressive pulmonary fibrosis.

We thank all the authors for their valuable contributions and the reviewers for their insightful feedback. We hope this Research Topic fosters further discussions and collaborative efforts to find reliable biomarkers for progressive pulmonary fibrosis.

